# Supplementary material for: Rational Screening of High-Voltage Electrolytes and Additives for Use in LiNi0.5Mn1.5O4-Based Li-Ion Batteries
Source: Molecules. 2022 Jun 3;27(11):3596. doi: 10.3390/molecules27113596 (PMC9182327; doi:10.3390/molecules27113596)
Supplement: Supplementary file 1 [file molecules-27-03596-s001.zip › molecules-1700336-supplementary.pdf]

Supplementary materials for

Rational Screening of High-Voltage Electrolytes and Additives for Use in LiNi0.5Mn1.5O4-Based Li-Ion Batteries

Oleg A. Drozhzhin [1,*], Vitalii A. Shevchenko [1,2], Zoia V. Bobyleva [3], Anastasia M. Alekseeva [1], and Evgeny V. Antipov [1,2]

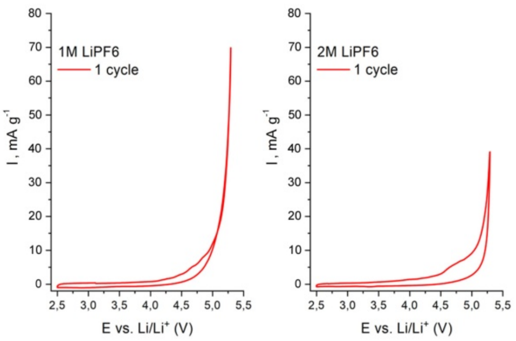

Figure S1. Results of cyclic voltammetry on "idle" electrodes for $LiPF_6$-based solutions in EC:DEC:DMC = 1:1:1 as a solvent with different salt concentrations.

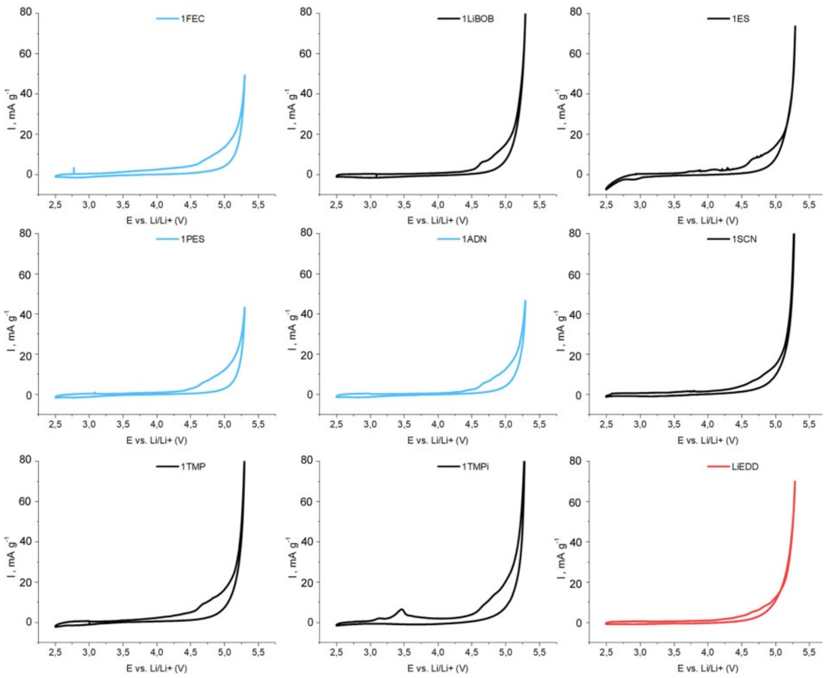

Figure S2. The results of cyclic voltammetry on "idle" electrodes for solutions based on 1M $LiPF_6$ in EC:DEC:DMC = 1:1:1 with various types of additives (blue color indicates electrolytes with improved anodic stability; black indicates electrolytes where the oxidation current at 5.3 is higher than in "standard" electrolyte; the latter is shown in red).

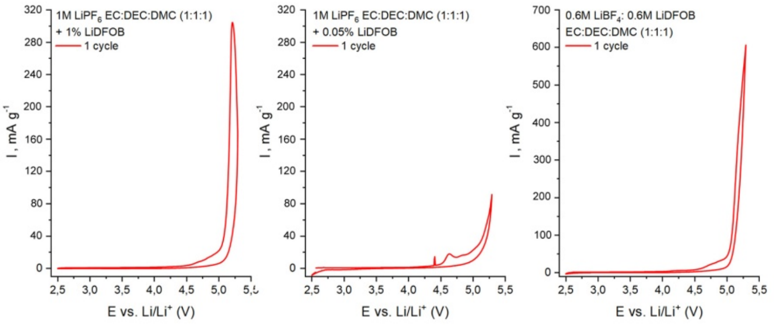

Figure S3. Results of cyclic voltammetry on "idle" electrodes for solutions with the addition of LiDFOB.

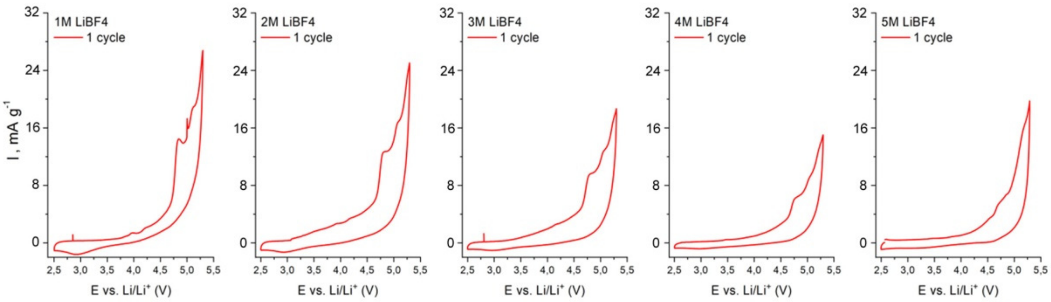

Figure S4. Results of cyclic voltammetry on "idle" electrodes for solutions based on $LiBF_4$ in SL with different salt concentrations.

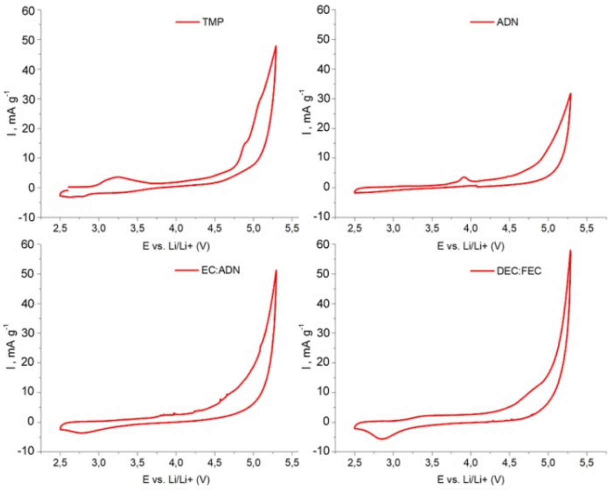

Figure S5. - Results of cyclic voltammetry on "idle" electrodes for solutions based on $LiBF_4$ in various solvents.

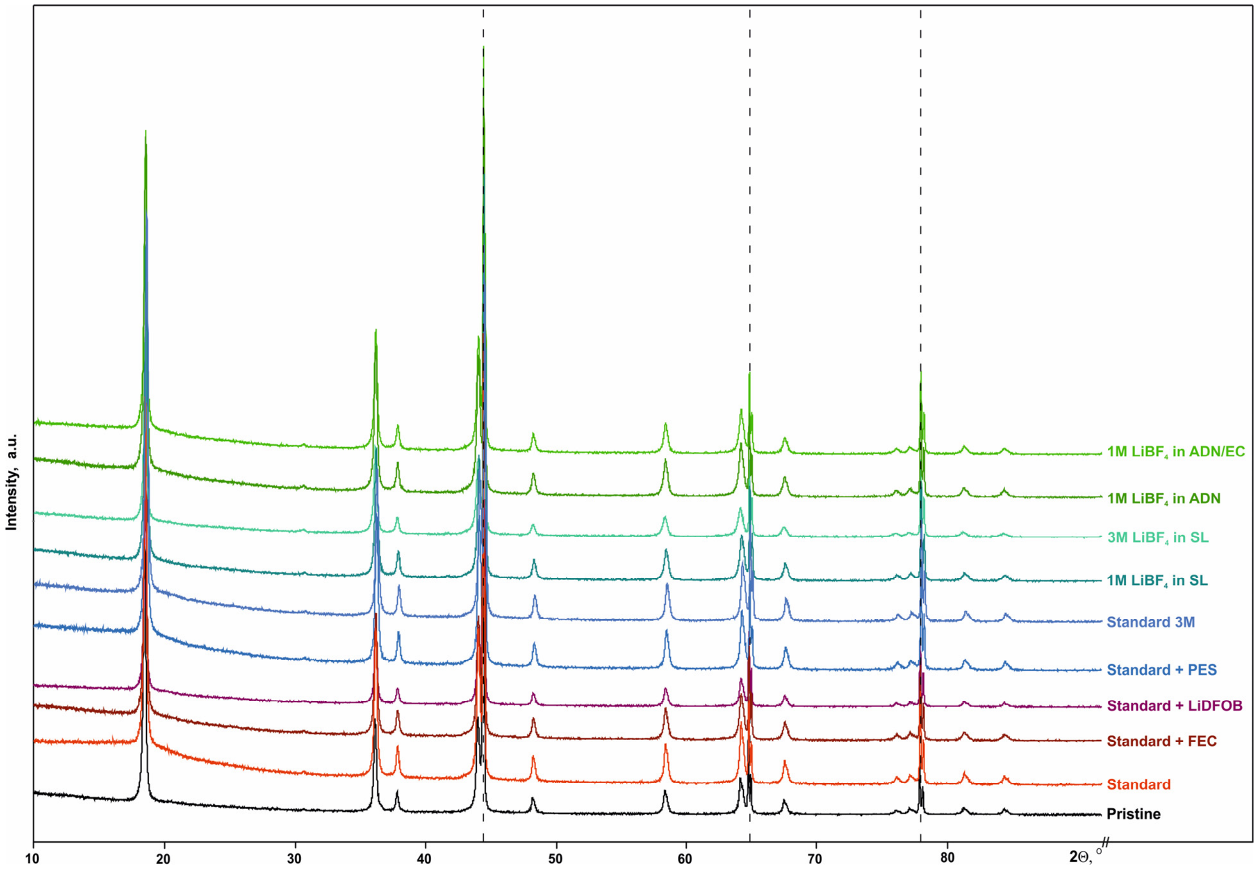

Figure S6. *Ex situ* PXRD patterns obtained for the pristine $LiNi_{0.5}Mn_{1.5}O_4$ cathode and for the cathodes after 140 charge-discharge cycles in selected electrolytes. The dotted lines mark Al current collector diffraction maxima.

Table S1. EDX data for the initial $LiNi_{0.5}Mn_{1.5}O_4$ electrode.

Spectrum	In stats.	O	F	S	Mn	Ni
Spectrum 1	Yes	46.21	23.80	0.60	22.61	6.79
Spectrum 2	Yes	44.49	25.79	0.21	22.44	7.07
Spectrum 3	Yes	49.26	19.66	0.98	22.88	7.23
Spectrum 4	Yes	47.88	21.57	0.39	23.09	7.08
Spectrum 5	Yes	47.36	21.04	0.18	23.84	7.58
Spectrum 6	Yes	45.82	22.81	0.16	23.82	7.38
Spectrum 7	Yes	47.97	17.85	1.12	25.07	7.99
Spectrum 8	Yes	48.55	19.04	0.17	24.51	7.72
Spectrum 9	Yes	48.75	20.12	0.07	23.30	7.75
Spectrum 10	Yes	51.68	16.71	0.97	23.12	7.52
Mean		47.80	20.84	0.49	23.47	7.41
Std. deviation		2.01	2.77	0.40	0.84	0.37

Table S2. EDX data for the $LiNi_{0.5}Mn_{1.5}O_4$ electrode cycled in “Standard” electrolyte.

Spectrum	In stats.	O	F	P	Mn	Ni
Spectrum 1	Yes	29.76	45.17	1.92	17.52	5.62
Spectrum 2	Yes	28.16	47.97	1.84	16.87	5.15
Spectrum 3	Yes	32.69	44.30	1.75	16.05	5.22
Spectrum 4	Yes	30.29	44.05	1.51	18.31	5.83
Spectrum 5	Yes	29.77	46.46	1.51	17.00	5.26
Mean		30.13	45.59	1.71	17.15	5.42
Std. deviation		1.64	1.63	0.19	0.84	0.29

Table S3. EDX data for the $LiNi_{0.5}Mn_{1.5}O_4$ electrode cycled in “Standard+FEC” electrolyte.

Spectrum	In stats.	O	F	Si	P	Mn	Ni

Spectrum 1	Yes	23.86	55.25	0.62	1.84	13.91	4.50
Spectrum 2	Yes	28.88	47.72	0.56	1.95	15.74	5.15
Spectrum 3	Yes	23.88	55.57	1.09	1.75	13.31	4.39
Spectrum 4	Yes	22.01	58.54	1.47	1.36	12.62	4.00
Spectrum 5	Yes	24.16	54.02	0.80	1.86	14.51	4.65
Mean		24.56	54.22	0.91	1.75	14.02	4.54
Std. deviation		2.56	4.00	0.38	0.23	1.19	0.42

Table S4. EDX data for the $LiNi_{0.5}Mn_{1.5}O_4$ electrode cycled in "Standard+LiDFOB" electrolyte.

Spectrum	In stats.	O	F	Si	P	Mn	Ni
Spectrum 1	Yes	26.94	50.22	1.52	0.93	15.37	5.02
Spectrum 2	Yes	22.99	54.82	1.50	0.95	15.04	4.70
Spectrum 3	Yes	28.51	49.16	1.67	0.87	15.03	4.77
Spectrum 4	Yes	25.55	51.91	1.44	0.78	15.56	4.76
Spectrum 5	Yes	25.52	54.00	1.24	0.84	14.09	4.31
Mean		25.90	52.02	1.47	0.87	15.02	4.71
Std. deviation		2.04	2.41	0.15	0.07	0.57	0.25

Table S5. EDX data for the $LiNi_{0.5}Mn_{1.5}O_4$ electrode cycled in "Standard+PES" electrolyte.

Spectrum	In stats.	O	F	Si	P	Mn	Ni
Spectrum 1	Yes	18.14	65.87	1.54	1.56	10.04	2.85
Spectrum 2	Yes	20.60	62.75	1.66	1.45	10.28	3.27
Spectrum 3	Yes	16.94	67.05	1.82	1.63	9.56	3.00
Spectrum 4	Yes	16.53	67.93	1.91	1.30	9.51	2.81
Spectrum 5	Yes	18.53	64.52	1.08	1.56	10.93	3.39
Mean		18.15	65.62	1.60	1.50	10.06	3.06
Std. deviation		1.60	2.06	0.32	0.13	0.58	0.26

Table S6. EDX data for the $LiNi_{0.5}Mn_{1.5}O_4$ electrode cycled in "Standard 3M" electrolyte.

Spectrum	In stats.	O	F	P	Mn	Ni
Spectrum 1	Yes	22.02	55.80	1.25	15.87	5.05
Spectrum 2	Yes	21.80	55.35	1.50	15.95	5.40
Spectrum 3	Yes	21.13	55.52	1.86	16.42	5.07
Spectrum 4	Yes	25.64	51.59	1.16	16.33	5.29
Spectrum 5	Yes	22.36	54.43	1.42	16.66	5.13
Mean		22.59	54.54	1.44	16.25	5.19
Std. deviation		1.76	1.73	0.27	0.33	0.15

Table S7. EDX data for the $LiNi_{0.5}Mn_{1.5}O_4$ electrode cycled in "1M $LiBF_4$ in SL" electrolyte.

Spectrum	In stats.	O	F	S	Mn	Ni
Spectrum 1	Yes	44.51	23.90	0.87	23.67	7.05
Spectrum 2	Yes	47.11	21.96	0.74	23.38	6.82
Spectrum 3	Yes	47.53	21.20	1.05	22.98	7.24
Spectrum 4	Yes	41.45	27.72	0.76	23.16	6.90
Spectrum 5	Yes	43.63	24.48	0.70	23.83	7.36
Mean		44.85	23.85	0.83	23.40	7.07
Std. deviation		2.52	2.55	0.14	0.35	0.23

Table S8. EDX data for the $LiNi_{0.5}Mn_{1.5}O_4$ electrode cycled in "3M $LiBF_4$ in SL" electrolyte.

Spectrum	In stats.	O	F	S	Mn	Ni
Spectrum 1	Yes	34.83	34.15	0.78	22.45	7.78
Spectrum 2	Yes	39.41	29.73	0.76	22.45	7.66
Spectrum 3	Yes	38.41	30.94	0.85	22.15	7.65
Spectrum 4	Yes	40.60	28.19	0.79	22.65	7.78
Spectrum 5	Yes	39.71	29.71	0.69	22.59	7.31
Mean		38.59	30.54	0.77	22.46	7.64
Std. deviation		2.24	2.24	0.06	0.19	0.19

Table S9. EDX data for the $LiNi_{0.5}Mn_{1.5}O_4$ electrode cycled in "1M $LiBF_4$ in ADN" electrolyte.

Spectrum	In stats.	O	F	Mn	Ni
Spectrum 1	Yes	40.64	25.40	25.78	8.18
Spectrum 2	Yes	42.73	24.25	24.96	8.06
Spectrum 3	Yes	37.13	29.41	25.01	8.44
Spectrum 4	Yes	37.86	27.18	26.91	8.06
Spectrum 5	Yes	40.31	25.23	26.22	8.24
Mean		39.73	26.30	25.78	8.20

Table S10. EDX data for the $LiNi_{0.5}Mn_{1.5}O_4$ electrode cycled in "1M $LiBF_4$ in AND/EC" electrolyte.

Spectrum	In stats.	O	F	Mn	Ni
Spectrum 1	Yes	34.01	34.00	24.04	7.95
Spectrum 2	Yes	36.71	28.90	25.89	8.49
Spectrum 3	Yes	40.26	26.28	24.88	8.59
Spectrum 4	Yes	31.08	37.56	23.55	7.81
Spectrum 5	Yes	32.48	35.15	24.65	7.72
Mean		34.91	32.38	24.60	8.11
Std. deviation		3.65	4.65	0.89	0.40
